# Single-Cell RNA Sequencing and Quantitative Proteomics Analysis Elucidate Marker Genes and Molecular Mechanisms in Hypoplastic Left Heart Patients With Heart Failure

**DOI:** 10.3389/fcell.2021.617853

**Published:** 2021-02-25

**Authors:** Li Ma, Na Zhou, Rongjun Zou, Wanting Shi, Yuanyuan Luo, Na Du, Jing Zhong, Xiaodong Zhao, Xinxin Chen, Huimin Xia, Yueheng Wu

**Affiliations:** ^1^The First Affiliated Hospital of Jinan University, Guangzhou, China; ^2^Heart Center, Guangzhou Women and Children’s Medical Center, Guangzhou Medical University, Guangzhou, China; ^3^Guangdong Provincial Key Laboratory of Research in Structural Birth Defect Disease, Department of Pediatric Surgery, Guangzhou Women and Children’s Medical Center, Guangzhou Medical University, Guangzhou, China; ^4^Department of Paediatrics, Guangzhou Women and Children’s Medical Center, Guangzhou Medical University, Guangzhou, China; ^5^Department of Surgical Nursing, Guangzhou Women and Children’s Medical Center, Guangzhou Medical University, Guangzhou, China; ^6^Department of Cardiovascular Surgery, Guangdong Cardiovascular Institute, Guangzhou, China; ^7^Guangdong Provincial Key Laboratory of South China Structural Heart Disease, Guangdong Provincial People’s Hospital and Guangdong Academy of Medical Sciences, School of Medicine, South China University of Technology, Guangzhou, China

**Keywords:** hypoplastic left heart, single-cell RNA sequencing, quantitative proteomics analysis, cardiac development, hub genes, heart failure

## Abstract

**Objective:**

To probe markers and molecular mechanisms of the hypoplastic left heart (HLH) by single-cell RNA sequencing (scRNA-seq) and quantitative proteomics analysis.

**Methods:**

Following data preprocessing, scRNA-seq data of pluripotent stem cell (iPSC)-derived cardiomyocytes from one HLH patient and one control were analyzed by the Seurat package in R. Cell clusters were characterized, which was followed by a *pseudotime* analysis. Markers in the *pseudotime* analysis were utilized for functional enrichment analysis. Quantitative proteomics analysis was based on peripheral blood samples from HLH patients without heart failure (HLH-NHF), HLH patients with heart failure (HLH-HF), and healthy controls. Hub genes were identified by the intersection of *pseudotime* markers and differentially expressed proteins (DE-proteins), which were validated in the GSE77798 dataset, RT-qPCR, and western blot.

**Results:**

Cardiomyocytes derived from iPSCs were clustered into mesenchymal stem cells, myocardium, and fibroblast cells. *Pseudotime* analysis revealed their differentiation trajectory. Markers in the three *pseudotime* clusters were significantly associated with distinct biological processes and pathways. Finally, three hub genes (MMP2, B2M, and COL5A1) were identified, which were highly expressed in the left (LV) and right (RV) ventricles of HLH patients compared with controls. Furthermore, higher expression levels were detected in HLH patients with or without HF than in controls.

**Conclusion:**

Our findings elucidate marker genes and molecular mechanisms of HLH, deepening the understanding of the pathogenesis of HLH.

## Introduction

Hypoplastic left heart (HLH) is a rare and complex form of congenital heart disease (CHD) including a spectrum of malformations that are characterized by the dysplasia of the left-sided cardiac structures, and thus resulting in the obstruction of the flow of blood into the systemic circulation (Tchervenkov et al., 2006). Epidemiological Investigation of HLH defect that accounts for 2–9% of all infants born with CHD (Hoffman and Kaplan, 2006; Bittle et al., 2018). The prevalence of HLH is approximately 2–3 cases per 10,000 live births (Gordon et al., 2008; Reller et al., 2008; Fixler et al., 2010). HLH accounts for 2–4% of congenital heart malformation-related deaths among newborns (Alphonso et al., 2020). Without intervention, this disease is highly fatal and remains clinically challenging, with approximately 25% of deaths in the first few weeks of life (Egbe et al., 2015). Despite recent advances in multistage reconstructive Norwood or Norwood-like operation, transferring the systemic circulation based on right ventricle (RV) physiology, approximately one-third of HLH patients die from terminal RV failure by the age of 25 (Bittle et al., 2018). Currently, there are two main treatments: heart transplantation and staged functional univentricular palliation (Barron et al., 2009). Heart transplantation is the sole treatment strategy for end-stage HF patients, accompanied by long-term immunosuppression and graft dysfunction (Everitt et al., 2012). The morbidity and mortality of HLH patients are determined by the function of the tricuspid valve and RV (Carrillo et al., 2020). Despite advances in the 3-stage surgery and medication approach, HLH still has a high mortality.

Human genetic studies suggest that HLH has genetic heterogeneity and polygenicity in etiology (Yagi et al., 2018). Genetic linkage research has demonstrated many important loci in the relatives of HLH, confirming genetic heterogeneity as well as common etiology (Martin et al., 2007; Hinton et al., 2009; McBride et al., 2009). Despite large-scale chromosomal abnormalities that have been detected among a high proportion of patients with extracardiac defects, most cases of patients scarcely exhibit large-scale alterations with other phenotypes (Glidewell et al., 2015). Furthermore, the genetic changes related to the etiology of non-syndromic cases remain unclear. Increasing evidence suggests that a single gene mutation could contribute to the progression of HLH. For example, mutations in NKX2-5 (Elliott et al., 2003), NOTCH1 receptor (Hrstka et al., 2017), and the MYH6 head domain variant (Kim et al., 2020) have been detected in HLH patients. Furthermore, copy number variants (CNVs) can affect the transcription of cardiovascular genes, thereby affecting the etiology of HLH (Glidewell et al., 2015). Researchers try to interpret genetic effect or molecular regulation mechanism in the fields of HLH occurrence, development and complications, and so on. But most studies, undeniably, ignore the clarification of the morphogenetic background and correlation analysis among between gene-features (Anderson).

scRNA-seq can simultaneously study the expression levels of multiple genes in thousands of single cells. It has been widely used to identify various cell types in the pathological process of cardiovascular diseases (Chaudhry et al., 2019). Furthermore, scRNA-seq has been able to explore distinct heterogeneity within a population of single-cell subtypes. Characterization of phenotypes for individual cells based on transcriptome data can depict cell development and expound changes in specific subgroups due to diseases. Quantitative proteomics is one of the most important applications in the field of proteomics research. Through quantitative proteomics technology, the changes in protein levels between samples can be quantitatively compared, which has been applied to cardiovascular disease etiology (Motiwala, 2019). In this study, we identified proteins related to HLH progression based on scRNA-seq of cardiomyocytes derived from iPSCs and quantitative proteomics analysis of HLH blood samples (Paige et al., 2020), which may provide evidence of HLH molecular mechanisms.

## Materials and Methods

### scRNA-Seq Data

This study obtained single-cell transcriptome profiles of cardiomyocytes that were derived from induced pluripotent stem cells (iPSCs) isolated from one HLH patient and one control who experienced cardiac-directed differentiation from the GSE146763 dataset in the Gene Expression Omnibus (GEO^1^) repository (Barrett et al., 2013; Miao et al., 2020).

### Data Preprocessing

10x Genomics data were read by the Seurat package in R, version 3.1 (Hafemeister and Satija, 2019). Cells with >2500 or <200 genes or a single cell with more than 5% mitochondrial genes were removed. Then, data were normalized by the LogNormalize function. Using the FindVariableFeatures function, the mean and variance of each gene were calculated. Highly variable genes between different cells were obtained for downstream analysis. The data were scaled through the ScaleData function. Dimensionality reduction analysis was then presented based on principal component analysis (PCA). The cells with similar expression were clustered through the FindClusters function. The shared nearest neighbor (SNN) was utilized to identify cell types. First, the SNN graph was constructed through k nearest neighbors, and then the module was optimized through the Louvain algorithm to determine the clustering results (Hafemeister and Satija, 2019). The resolution was set as 1.0. The cell clusters were visualized by t-distributed stochastic neighbor embedding (t-SNE).

### Differential Expression Analysis

Differential expression analysis was performed to find marker genes for different cell clusters through the FindMarkers function, with the threshold of the percentage of any gene detected in the two cell populations ≥ 0.25 and log fold change (FC) ≥ 0.25 (Hafemeister and Satija, 2019). Using the Doheatmap function, a heat map was visualized for the indicated cells and marker genes. Each cell cluster displayed the top 20 marker genes.

### *Pseudotim*e Analysis

The objects in the Seurat were converted into those recognized by the Monocle2 package in R. First, following data normalization, feature genes that defined a cell’s progress were chosen. Then, data dimensionality was reduced with the DDRTree method, followed by cell order along the trajectory. *Pseudotime* marker gene sets were obtained by the differential GeneTest function (Hafemeister and Satija, 2019). The root state parameter was utilized to specify the starting end by the orderCells function. The branch that contained the most cells at state 0 was then identified.

### Functional Enrichment Analysis

To explore the potential biological functions of *pseudotime* marker genes, Gene Ontology (GO) and Kyoto Encyclopedia of Genes and Genomes (KEGG) enrichment analyses were carried out. GO annotation analysis was presented through the “*clusterprofiler*” package, including biological process, cellular component, and molecular function. An adjusted *p*-value < 0.05 was significantly enriched (Yu et al., 2012). KEGG pathway enrichment analysis was carried out based on the DAVID online database with the criteria of overlap ≥ 3, *p*-value < 0.05, and enrichment ≥ 1.5 (Dennis et al., 2003).

### Clinical Sample Collection

Peripheral blood samples from one HLH child without heart failure (HLH-NHF), one HLH child with heart failure (HLH-HF), and one healthy child (control) were collected from Guangzhou Women and Children’s Medical Center between June 1, 2018, and January 1, 2020. This research strictly followed the guidelines of the Declaration of Helsinki. All children were diagnosed by physical signs, two-dimensional echocardiogram (2-DE), electrocardiogram (ECG), cardiac ultrasonography, or cardiac CT that abided by the guidelines of The European Association for Cardio-Thoracic Surgery (EACTS). And the cases combined with ventricular septal defects (VSDs), atrial septal defects (ASDs), and double outlet right ventricle (DORV) were excluded. The diagnosis of heart failure was referred to the American Heart Association Foundation (ACCF)/American Heart Association (AHA) guideline (Yancy Cw Fau - Jessup et al.). The parents of all children signed written informed consent forms. This study was approved by the Ethics Committee of the Chinese Clinical Trial Registry Center and Guangzhou Women and Children’s Medical Center (Registration number: ChiCTR-EOC-17013273; Approved No. of ethics committee: 2017103101).

### Mass Spectrometry Analysis

After centrifugation at 12,000 *g*, the supernatant of blood samples was harvested and stored at –80°C until further analysis. The samples were incubated with a reaction solution composed of 1% SDC, 10 mM TCEP, and 40 mM CAA at 60°C for 30 min for protein denaturation, disulfide bond reduction, and sulfhydryl alkylation. The protein concentration was determined by the Bradford method. The sodium deoxycholate (SDC) concentration was diluted to <0.5%, and trypsin was added according to a 1:50 mass ratio of enzyme to protein, followed by incubation at 37°C with shaking overnight for digestion. The next day, trifluoroacetate (TFA) was added to terminate the digestion. The pH of the solution was adjusted to approximately 6.0. Following centrifugation at 12,000 × *g* for 15 min, the supernatant was collected for desalination. After the desalted peptide solution was drained by a centrifugal concentrator, the samples were stored at –20°C for testing.

Using data-independent acquisition (DIA) technology, mass spectrometry analysis occurred through a combined system of Ultimate3000 (capillary flow) and Q Exactive HFX (Gillet et al., 2012). The prepared peptide sample was first bound to the Trap column. After that, it was separated by an analytical column (300 μm × 150 mm, 2 μm particle size, 100 Å pore size, Acclaim PepMap C18 column, Thermo). The two mobile phases for establishing an analytical gradient were buffered A-0.1% (V/V) formic acid, 2% ACN, 3% DMSO in H_2_O, buffer B-0.1% (V/V) formic acid and 3% DMSO in acetonitrile. During SWATH scanning (Gillet et al., 2012), each cycle of scanning included one MS1 scan (scanning range 350–1,250 m/z, resolution 60 K, AGC 3e6, Max. IT 20 ms) and 40 variable window MS2 scans (resolution 30 K, AGC 1e6, Max. IT auto).

### DIA Quantitative Proteomics Analysis

The mass spectrum files scanned by DIA were processed by DIA-Umpire to obtain secondary mass spectrum files that can be used for database search. MSFragger software was utilized to search the database of the secondary mass spectrum, and the result obtained was used as a spectral library for subsequent targeted extraction. The DIA-NN algorithm was used for DIA targeted extraction and quantification (Demichev et al., 2020). The screening criteria were as follows: false discovery rate (FDR) < 0.01. The protein quantitative intensity data obtained by DIA analysis were converted by log2. Moreover, data were filled using the imputation algorithm in Perseus software. Following normalization, differential expression analysis was performed by Student’s *t*-test. DEproteins were screened with the threshold of FC ≥ 2 or ≤0.5 and *p*-value ≤ 0.05. Then, GO, KEGG, and Clusters of Orthologous Groups (COG^2^) annotation and enrichment analyses were performed. An adjusted *p*-value < 0.05 was significantly enriched. The RAW data and analysis scripts can be accessed from Dr. RZ upon request.

### Protein–Protein Interaction (PPI) Network

Through the STRING (version 11.0^3^) database (Szklarczyk et al., 2017), PPI networks were constructed based on the DEproteins in the three groups of HLH-NHF vs. control, HLH-HF vs. control, and HLH-NHF vs. HLH-HF.

### Validation by RNA-Seq

RNA-seq profiles of HLH-LV and HLH-RV from HLH mice and their littermate controls (control-LV and control-RV) were retrieved from the GSE77798 dataset in the GEO database^4^ (Liu et al., 2017). The expression of MMP2, COL5A1, and B2M was verified in the control-LV vs. HLH-LV and control-RV vs. HLH-RV groups.

### Real-Time Quantitative Polymerase Chain Reaction (RT-qPCR) Assay

According to the manufacturer’s manual, TRIzol reagent was used to extract total RNA from blood samples. After centrifugation at 12,000 *g* for 5 min at 4°C, the supernatant was collected. The concentration and purity of the samples were determined by measuring the absorbance at 260 and 280 nm wavelengths with a spectrophotometer. Total RNA was reverse transcribed into cDNA by a reverse transcription kit (Takara, Dalian, China). RT-qPCR was performed on an Applied Biosystems 7500 Real-Time PCR System (Applied Biosystems, Foster City, CA, United States) using an SYBR Green PCR kit (Takara). The primer sequences of the target genes were as follows: MMP2 (forward: 5′−ATACCATCGAGACCATGCG-3′; reverse: 5′−CCAATGATCCTGTATGTGATCTG-3′), COL5A1 (forward: 5′−ATTGAGCAGATGAAACGGC-3′; reverse: 5′−A GTATTCACCATCTGGGAAGTC-3′), and B2M (forward: 5′−T GTACTACACTGAATTCACCC-3′; reverse: 5′−CTTACATGTC TCGATCCCAC-3′). GAPDH was used as an internal reference. Relative expression levels were calculated with the 2^−ΔΔCt^ method.

### Western Blot

Proteins were extracted from blood samples, and their concentration was determined using a BCA kit (Beyotime, Shanghai, China). Extracted proteins were separated using an SDS-PAGE separation gel, followed by transference onto membranes. After being blocked with skimmed milk for 1 h, the membrane was incubated with primary antibodies against MMP2, COL5A1, B2M, and GAPDH at 4°C overnight, followed by incubation with secondary antibodies for 1 h. The antibodies used were from Cell Signaling Technology (Danvers, MA, United States). Immunoluminescence agents (Pierce, Rockford, IL, United States) were added to the membrane. The protein blots were visualized on Kodak Scientific Imaging Systems (New Haven, CT, United States).

### Statistical Analysis

Statistical analysis was p using R language (version 3.6.3^5^) and GraphPad Prism software (version 8.0). For RT-qPCR and western blot assays, each experiment was repeated ≥ 3 times. Data are expressed as the mean ± standard deviation. Multiple comparisons were analyzed by one-way analysis of variance (ANOVA). *P* < 0.05 was statistically significant.

## Results

### Characterization of Cell Cluster Compositions in HLH and Normal Cardiomyocytes Derived From iPSCs

**FIGURE 1:**
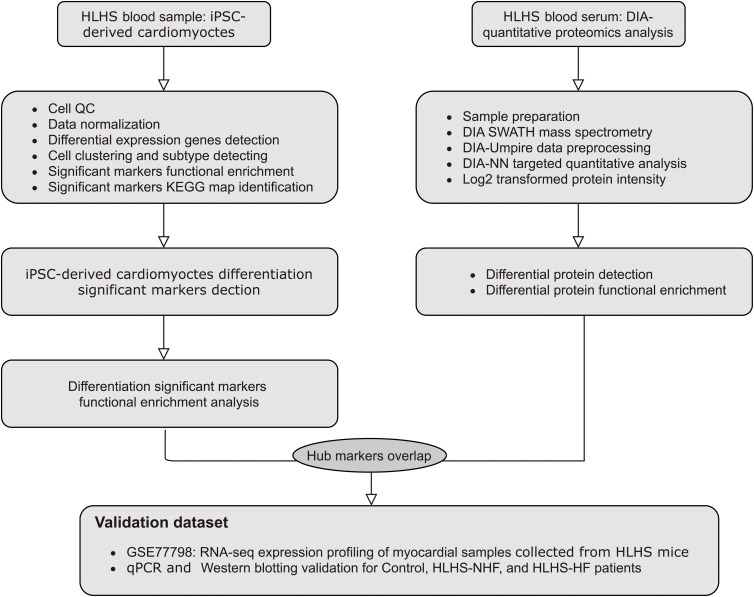
The workflow of this study.

**FIGURE 2:**
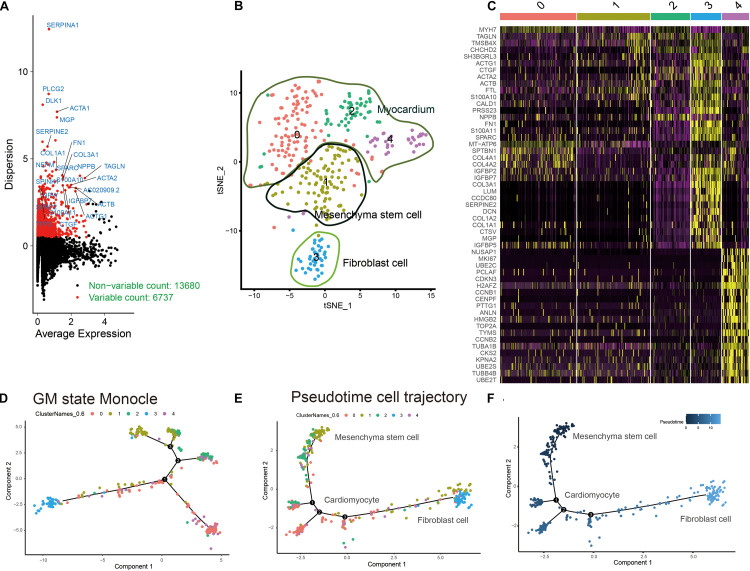
Characterization of cell cluster compositions in HLH and normal cardiomyocytes derived from iPSCs. **(A)** Identification of highly variable genes between different cardiomyocytes. Red dots indicate highly variable genes, and black dots indicate non-variable genes. **(B)** The *t*-SNE plot of cardiomyocyte populations. **(C)** Visualization of the top 20 marker genes for different cell clusters. **(D–F)** *Pseudotime* analysis results. Different colors express different states of cell clusters. The shade of the color indicates the sorting of the cells according to the *pseudotime* value. Each dot represents a cell, and cells with similar states are clustered together. Each branch point represents a decision point of a possible biological process.

The workflow of this research is shown in Figure 1. A total of 6737 highly variable genes between different HLH and normal cardiomyocytes derived from iPSCs were screened for downstream analysis, which could highlight the biosignals in the scRNA-seq dataset (Figure 2A). Based on HLH and normal cardiomyocytes derived from iPSCs, a total of five cell clusters (0–4) were clustered (Figure 2B). Clusters 0, 2, and 4 were categorized as the myocardium population. Cluster 1 was classified as the mesenchymal stem cell population. Cluster 3 was composed of fibroblast cells. Following identification of the cell clusters, marker genes for these cell clusters were screened with the criteria of min.pct = 0.25 and log FC ≥ 0.25 (Figure 2C). The top 20 marker genes are displayed for each cell cluster. Then, we analyzed the state of the four cell clusters. *Pseudotime* analysis revealed their differentiation trajectory (Figures 2D–F).

### Functional Enrichment Analysis of Marker Genes in *Pseudotime* Clusters

**FIGURE 3:**
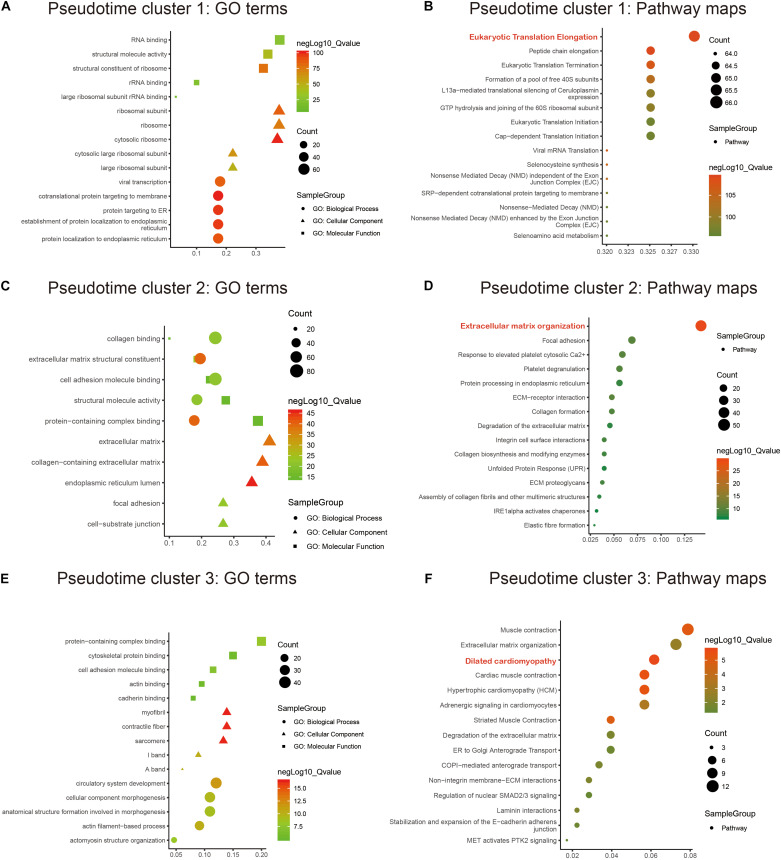
Functional enrichment analysis of marker genes in the *pseudotime* clusters. GO and KEGG annotation enrichment analyses of marker genes in *pseudotime* cluster 1 **(A,B)**, *pseudotime* cluster 2 **(C,D)**, and *pseudotime* cluster 3 **(E,F)**. GO includes biological processes (circle), cellular components (triangle), and molecular functions (rectangle).

GO and KEGG annotation enrichment analyses were presented to explore the biological functions of cell development-related marker genes from *pseudotime* analysis. For *pseudotime* cluster 1, marker genes distinctly exhibited RNA binding functions and were significantly associated with protein localization to the endoplasmic reticulum (Figure 3A) and the eukaryotic translation elongation pathway (Figure 3B). Marker genes in *pseudotime* cluster 2 had collagen binding, extracellular structural constituent, and cell adhesion molecule binding functions (Figure 3C) and were distinctly enriched in the extracellular matrix organization pathway (Figure 3D). Marker genes in *pseudotime* cluster 3 were significantly related to circulatory system development (Figure 3E) and dilated cardiomyopathy (Figure 3F).

### DIA Quantitative Proteomics Analysis of HLH-NHF, HLH-HF, and Control Blood Samples

**FIGURE 4:**
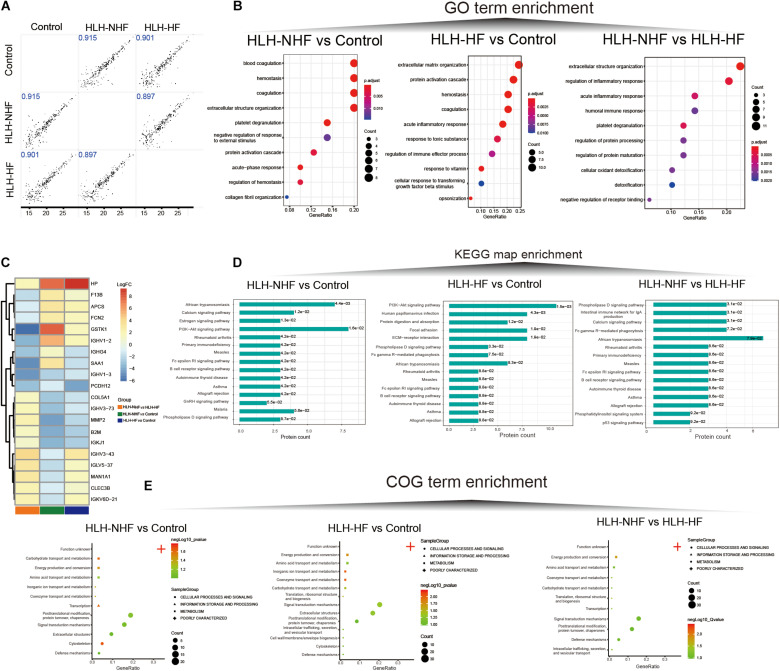
Data-independent acquisition quantitative proteomics analysis of HLH-NHF, HLH-HF, and control plasma samples. **(A)** Correlation analysis of plasma proteins between the three groups of HLH-NHF, HLH-HF, and control samples. **(B)** The top 10 GO term enrichment analysis results of DEproteins in the three groups of HLH-NHF vs. control, HLH-HF vs. control, HLH-NHF vs. HLH-HF. **(C)** A heat map visualizing the top 20 DEproteins in the three groups. **(D)** The top 15 KEGG map enrichment analysis results of DEproteins in the three groups. **(E)** The COG term enrichment analysis results of DEproteins in the three groups.

Data-independent acquisition quantitative proteomics was analyzed based on HLH-NHF, HLH-HF, and control blood samples. The sample information and clinical features are presented in Supplementary Table 1. In total, 276 plasma proteins were obtained. There were high correlations of plasma proteins between HLH-NHF, HLH-HF, and control samples (Figure 4A). With the threshold of FC ≥ 2 or ≤0.5 and *p*-value ≤ 0.05, we identified 65 DEproteins (36 upregulated and 29 downregulated) between HLH-NHF and the control, 84 DEproteins (46 upregulated and 38 downregulated) between HLH-HF and the control, and 85 DEproteins (48 upregulated and 37 downregulated) between HLH-NHF and HLH-HF. The GO enrichment analysis results showed that DEproteins in different comparison groups were enriched in distinct biological processes. In Figure 4B, DEproteins in HLH-NHF vs. control were significantly enriched in blood coagulation, hemostasis, and coagulation. DE proteins in HLH-HF vs. control mice were distinctly associated with extracellular matrix organization and the protein activation cascade. Moreover, DE proteins in HLH-NHF vs. HLH-HF had a significant relationship with extracellular structure organization and regulation of the inflammatory response. The heat maps visualized the top 20 plasma DEproteins in the three groups. As depicted in Figure 4C, there were distinct differences in their expression. The KEGG pathway enrichment analysis results revealed that the DEproteins were related to distinct signaling pathways. For instance, in Figure 4D, the HLH-NHF vs. control and HLH-HF vs. control groups had the PI3K-Akt signaling pathway distinctly enriched by DEproteins. In the HLH-NHF vs. HLH-HF group, DEproteins were significantly enriched in the phospholipase D and calcium signaling pathways. Furthermore, the COG term enrichment analysis results demonstrated that DEproteins in the three groups had a significant relationship with carbohydrate transport and metabolism, posttranslational modification, protein turnover, chaperones, energy production and conversion, and signal transduction mechanisms (Figure 4E).

### Validation of Hub Genes From scRNA-Seq and Proteomics for HLH

**FIGURE 5:**
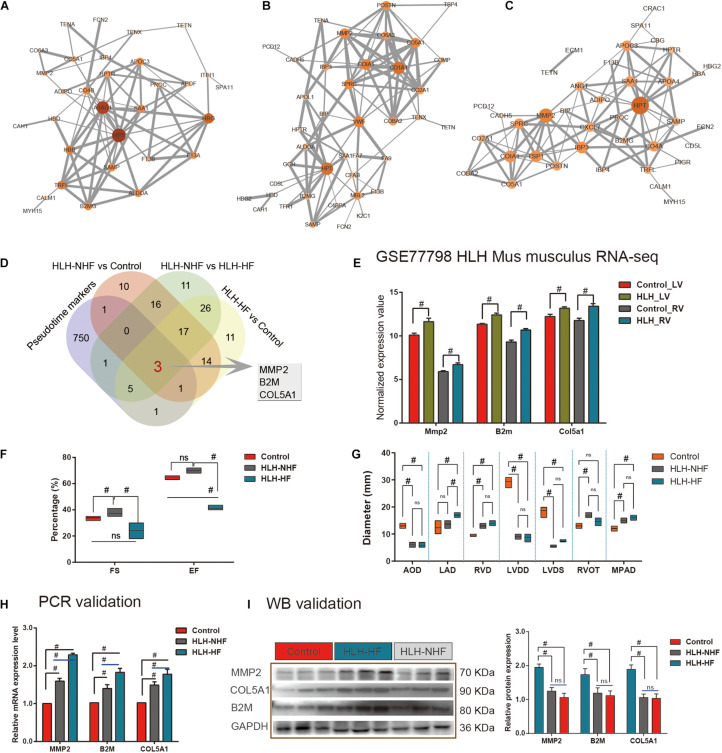
Validation of hub genes from scRNA-seq and proteomics for HLH. **(A)** A PPI network for DEproteins between HLH-NHF and the control. **(B)** A PPI network for DEproteins between HLH-HF and the control. **(C)** A PPI network for DEproteins between HLH-NHF and HLH-HF. Each node represents a DEprotein, and the lines between nodes represent interactions between DEproteins. The darker the color, the greater the fold change of a DEprotein. **(D)** Venn diagram visualizing three hub genes (MMP2, COL5A1, and B2M) by the intersection of *pseudotime* markers and DEproteins in HLH-NHF vs. control, HLH-HF vs. control, and HLH-NHF vs. HLH-HF. **(E)** Validation of the hub genes in the four groups of control LV, HLH-LV, control RV, and HLH-RV from the GSE77798 dataset. **(F)** Results of color Doppler echocardiography among the left ventricular ejection fraction (EF) and short rate of left ventricle short axis (FS). **(G)** The results of color Doppler echocardiography corresponding to the left ventricular size and main pulmonary artery diameter. **(H)** RT-qPCR validation of the hub genes in the control, HLH-NHF, and HLH-HF groups. **(I)** Western blot validation of the hub genes in the control, HLH-NHF, and HLH-HF groups. #*p* < 0.05 and ns, no statistical significance.

Protein–protein interaction networks were constructed to explore the interactions of DE proteins in the three groups of HLH-NHF vs. control (Figure 5A), HLH-HF vs. control (Figure 5B), and HLH-NHF vs. HLH-HF (Figure 5C). Among the three networks, HPT had the highest degree. Following combining *pseudotime* markers and DEproteins in HLH-NHF vs. control, HLH-HF vs. control, and HLH-NHF vs. HLH-HF, three hub genes were identified, including MMP2, COL5A1, and B2M (Figure 5D). Their expression was validated in the GSE77798 dataset. The results showed that MMP2, COL5A1, and B2M expression was significantly increased in the HLH-LV group compared to the control LV group (all *p*-values < 0.05; Figure 5E). Moreover, their expression was distinctly higher in the HLH-RV group than in the control RV group (all *p*-values < 0.05; Figure 5E).

Simultaneously, we obtained 6 cases of HLH and 3 cases of normal children’s peripheral blood samples from the biological sample bank of Guangzhou Women and Children’s Medical Center for verification. The corresponding clinical information was also collected. Among the six cases, includes three cases HLH-NHF and three cases HLH-HF, we found all the had a long history of patent foramen ovale (PFO), four (66.7%) suffered from pneumonia on admission, three (50%) with combined mitral and aortic atresia (MA/AA), two (33.3%) had mitral and aortic stenosis (MS/AS), and three (50%) had a long history of pulmonary hypertension (PH). Besides, the left ventricle (LV) ejection fraction (EF) and short rate of left ventricle short axis (FS) in HLH-HF patients were significantly reduced compared to those in the normal and HLH-NHF groups (all *p*-values < 0.05; Figure 5F). Additionally, as shown in Figure 5G, 2-DE provided the diameter size of left atrium diameter (LAD) in HLH-HF were significantly increased in compared with HLH-NHF (Mean Diff = –3.33; 95%CI of diff = –5.95 to –0.72; *p* < 0.05) and control group (Mean Diff = –4.67; 95%CI of diff = –7.28 to –2.05; *p* < 0.05), while no difference detected among the normal and HLH-NHF group (Mean Diff = –1.33; 95%CI of diff = –3.95 to 1.28). In contrast to the normal group, the diameter size of the left ventricular end-diastolic diameter (LVDD) was significantly decreased in HLH children (Control vs. HLH-NHF: Mean Diff = 20.33, 95%CI of diff = 17.72–22.95; Control vs. HLH-HF: Mean Diff = 20.67, 95%CI of diff = 18.05–23.28; all *p* < 0.05), as well as the size of left ventricular end-systolic diameter (LVDS; Control vs. HLH-NHF: Mean Diff = 13.33, 95%CI of diff = 10.72–15.95; Control vs. HLH-HF: Mean Diff = 11.33, 95%CI of diff = 8.721–13.95; all *p* < 0.05). In this study, all of the HLH cases had varying degrees of mitral valve dysplasia, includes atresia and stenosis, and showing a limitation in the dynamic activity of the stenotic valve. Additionally, the diameter size of RV (RVD) was apparently higher in HLH than in normal patients (Control vs. HLH-NHF: Mean Diff = –3.67, 95%CI of diff = –6.13 to –1.21; Control vs. HLH-HF: Mean Diff = –4.67, 95%CI of diff = –7.13 to –2.21; all *p* < 0.05). On the other hand, RV walls were hypertrophied in four HLH cases (66.7%), and the tricuspid annulus had a mild to moderate degree of dilation and regurgitation. In this setting, all of the HLH cases present aortic dysplasia, which is characterized by the decreased ascending aortic diameter (five cases, 83.3%) and the abnormalities of the descending aortic arch (two cases, 33.3%). In contrast to the normal group, the diameter size of aortic diameter (AOD) was significantly lower in HLH children (Control vs. HLH-NHF: Mean Diff = 7.00, 95%CI of diff = 4.54–9.46; Control vs. HLH-HF: Mean Diff = 7.17, 95%CI of diff = 4.71–9.63; all *p* < 0.05). No significant differences were detected among HLH-NHF and HLH-HF groups. Ultimately, the 2-DE results also shown a mild to moderate degree of MPAD dilatation in HLH cases than the normal group (Control vs. HLH-NHF: Mean Diff = –3.00, 95%CI of diff = –5.46 to –0.54; Control vs. HLH-HF: Mean Diff = –4.00, 95%CI of diff = –6.46 to –1.54; all *p* < 0.05).

We validated the expression of hub genes in blood samples from control, HLH-NHF, and HLH-HF patients by RT-qPCR and western blot. As shown in Figure 5F, compared to the control group, hub gene mRNA expression was markedly increased in both the HLH-NHF and HLH-HF groups (all *p*-values < 0.05; Figure 5H). There were distinctly higher mRNA expression levels in the HLH-HF group than in the HLH-NHF group (all *p*-values < 0.05). Consistently, western blot results showed that MMP2, COL5A1, and B2M exhibited higher protein expression levels in the HLH-NHF and HLH-HF groups than in the control group (all *p*-values < 0.05; Figure 5I). However, there was no significant difference in their expression between the HLH-NHF and HLH-HF groups (*p* > 0.05).

## Discussion

Hypoplastic left heart is one of the most complex congenital heart diseases and is usually established quickly based on symptoms, prenatal examination, and postnatal echocardiography. HLH is almost universally fatal among infants and children (Barron et al., 2009; Saraf et al., 2019). Remmell-Dow et al. (1995) compared 42 HLH hearts with 16 normal hearts and found eustachian valve dysplasia in up to 92.9% of HLH hearts, as well as 33.3% limbus dysplasia. Similarly, Bharati and Lev reported a surgical anatomy analysis of 230 HLH hearts that illustrated that 45.7% of hearts had aortic atresia (AS) and mitral stenos (MS), as well as 41.3% of hearts having aortic and mitral atresia (AA/MA) (Bharati et al., 1984). The same exciting research by Crucean et al. (2017) recategorized the HLH heart into the slit-like left ventricle (SLLV), miniaturized left ventricle (MLV), and thickened left ventricle with endocardial fibroelastosis (TLV-EFE) according to valve patency phenotype. In this study, they shared the proportion of each subtype of HLH that was up to 70% TLV-EFE, and there was no evidence of a connection between the degree of myocardial thickening and valvar stenosis (Crucean et al., 2017). Regarding anatomical heterogeneity, Stephens et al., (2020a,b) found that 30% of HLH combined with AA/MA, 22% had AS/MS, and 48% had MS/AA, and interestingly, these results showed that the syndrome was an acquired disease of fetal life, not abnormal embryogenesis.

Most patients still suffer from HF and recurrent pneumonia, although the treatment of the disease has been greatly improved until the Norwood operation or various surgical stages. Herein, the management and control of heart failure in HLH children are particularly important, while the causes of HLH are still unclear. The advancement of scRNA-seq and proteome technology opens a new chapter in the study of the pathological mechanism of HLH. For example, scRNA-seq studies in the adult heart have elucidated distinct differences in the gene expression of cardiomyocytes (Skelly et al., 2018). scRNA-seq can find more heterologous gene expression, which has not been detected by previous tissue-based RNA-seq (Chaudhry et al., 2019). In this study, we analyzed the transcriptional expression changes during the HLH development by the single-cell transcription profile. Furthermore, combined with DIA protein sequencing technology, the occurrence and progression of HLH were described from the two levels of transcription and protein expression. Three core regulatory factors of HLH were finally identified and validated by integrating scRNA-seq and DIA quantitative proteomics analysis, which could offer novel insights into the etiology of HLH.

In this study, we analyzed scRNA-seq data of cardiomyocytes derived from HLH and normal iPSCs. Three cell clusters, including mesenchymal stem cells, myocardium, and fibroblast cells, were characterized by identification of the specific expression of marker genes in each cell type, indicating distinct expression and functional differences in HLH iPSC-derived cardiomyocytes (Jiang et al., 2014). In many biological processes, cells are not completely synchronized. *Pseudotime* analysis was achieved by ranking cells based on the similarity of their gene expression profiles. During development, cells will respond to stimuli and change from one functional “state” to another throughout their life. Cells in different states express different genes and produce dynamic repetitive sequences of proteins and metabolites to complete their work. When a cell moves between different states, it undergoes a process of transcriptional recombination, in which some genes are silenced, and others are activated. Differential expression analysis was used to characterize cell clusters in this study. Compared with all other cells, differential expression analysis can identify gene sets that are more highly expressed in the cell cluster, which provides clues on the cell type/state or cell function in the cell cluster. Herein, we identified the genes that were regulated during the cell trajectory. GO and KEGG annotation and enrichment analysis revealed that these *pseudotime* marker genes in different clusters were involved in distinct key biological processes and pathways. For example, in *pseudotime* cluster 1, eukaryotic translation elongation was significantly enriched, indicating that these marker genes could be involved in mediating the cardiac translation process. Protein synthesis is a strictly regulated process. The increase in heart protein levels can induce cardiac hypertrophy and HF (Grund et al., 2019). Thus, the identification of genetic factors that regulate the level of protein synthesis in cardiomyocytes may offer promising therapeutic targets for HLH. Extracellular matrix organization was distinctly enriched by marker genes in *pseudotime* cluster 2, which was a critical factor for valve formation. It is also related to intrinsic endocardial defects in HLH (Miao et al., 2020). Furthermore, marker genes in *pseudotime* cluster 3 had a significant relationship with dilated cardiomyopathy, indicating that these genes might be important for heart development and cardiovascular disease (Sucharov et al., 2015).

Our DIA quantitative proteomics analysis of HLH-NHF, HLH-HF, and control blood samples identified 276 plasma proteins related to HLH. After the intersection of *pseudotime* markers and DEproteins in HLH-NHF vs. control, HLH-HF vs. control, and HLH-NHF vs. HLH-HF, three hub genes were identified, including MMP2, COL5A1, and B2M. We further validated their expression in an independent dataset. Our results demonstrated that they were expressed at low levels in both LV and RV HLH compared to controls. Moreover, our RT-qPCR and western blot results confirmed that they exhibited lower expression levels in HLH with or without HF than in controls. Cardiac fibrosis has been detected in HLH by histopathology (Kido et al., 2018). Increased angiotensin II and aldosterone levels promote collagen synthesis and reduce collagen breakdown by regulating matrix metalloproteinases, thereby inducing extracellular matrix (ECM) homeostasis (Nakano et al., 2017). Radosinska et al. (2017) found that ECM homeostasis was considered a structural and physiological foundation of the heart and that matrix metalloproteinases (MMPs) are an important environmental mediator of cardiac diastolic or systolic dysfunction (Radosinska et al., 2017). Moreover, both the MMP2 expression level in myocardial tissue and blood circulation was most frequently reported to represent myocardial remodeling accompanied by myofibrosis, inflammation, and consequent development of heart failure (George et al., 2005; Andreasová et al., 2020). It has been reported that COL5A1 mutations are closely related to the abnormal heart or arterial development (Weerakkody et al., 2016; Richer et al., 2020). Ramirez et al. (2012) identified that the expression level of COL5A1, reflecting the status of inflammatory and extracellular adhesion, was increased in left ventricle tissues in relation to the hypoxia response. Based on *in situ* hybridization analysis, Roulet’s group reported that the expression of COL5A1 plays an important role in the development of functional connective tissues, especially in the heart, dorsal aorta wall, skin, and vertebral column, suggesting that COL5A1 plays an important role in the development of these organs in developing embryos (Roulet et al., 2007). Interestingly, Yokota et al. (2020) found that the COL5A1 expression level significantly correlated with scar size after myocardial infarction mechanically, not only in the regulation of the size of cardiac scars but also in tissue mechanical properties in an integrin-dependent manner. The expression level of B2M in the plasma is not simply a significant marker for kidney injury but a novel regulator involved in heart failure and heart remodeling (Vianello et al., 2015). Vianello’s group demonstrated that the B2M level was increased in chronic heart failure patients and that it was positively correlated with the levels of proBNP and GFR but negative in percent ejection fraction (Vianello et al., 2015). Using 9,988 participants’ data from the Atherosclerosis Risk in Communities (ARIC) study, we showed a more sensitive advantage in predicting outcomes, including kidney function, cardiovascular disease, and mortality, in the general population for serum B2M levels (Astor et al., 2012). Through retrospective analysis of 460 elderly isolated systolic hypertension (ISH) patients, Wang’s group suggested that serum B2M level is an independent risk factor associated with major cardiovascular events and all-cause death (Wang et al., 2018). Thus, the three hub genes could participate in the pathogenesis of HLH and become promising therapeutic targets for HLH, which deserves more in-depth exploration in future research.

## Conclusion

Taken together, this study clarified the heterogeneity of HLH cardiomyocytes based on scRNA-seq expression profiles. Furthermore, we characterized the type and function of different cell clusters through marker gene or functional annotation analysis. The cell types or states were organized into a trajectory. After validation, three hub genes (MMP2, COL5A1, and B2M) were identified by combining quantitative proteomics analysis, which could become promising therapeutic targets for HLH. Our research offers new insights into the molecular mechanism of HLH pathogenesis, which provide a theoretical basis for new therapeutic interventions. Here, several limitations were also detected in our study. First, there is no systematic correlation analysis constructed among the clinical features and hub marker’s expression. Second, HLH is still controversial and debatable, and its definition happens to be constantly improved and supplemented, among which the most authoritative one is defined as the left side of the heart is underdeveloped, while excludes the aortic atresia with VSD and a well-developed mitral valve and LV. There are still shortcomings in this study that applied the term “HLH” not the common term “hypoplastic left heart syndrome (HLHS)” since the complexity of morphologic and physiologic characteristics in HLHS is hard to define based on clinical examination in our center. Third, our verification and quantitative proteomics analysis of hub gene expression are based on the HLH patient’s blood serum, not the myocardial samples, which has certain limitations.

## Data Availability Statement

The data presented in the study are deposited in the National Genomics Data Center (NGDC; https://bigd.big.ac.cn/omix/) repository, accession number (OMIX234; BioProject accession: PRJCA004354).

## Ethics Statement

The studies involving human participants were reviewed and approved by the patients of all children signed written informed consent. This study was approved by the Ethics Committee of Chinese Clinical Trial Registry Center and Guangzhou Women and Children’s Medical Center (Registration number: ChiCTR-EOC-17013273; Approved No. of ethic committee: 2017103101). Written informed consent to participate in this study was provided by the participants’ legal guardian/next of kin. Written informed consent was obtained from the individual(s), and minor(s)’ legal guardian/next of kin, for the publication of any potentially identifiable images or data included in this article.

## Author Contributions

LM, NZ, and RZ takes responsibility for all aspects of the reliability and freedom from bias of the data presented and their discussed interpretation, drafting the article. WS, YL, ND, JZ, and XZ takes responsibility for statistical analyses, and interpretation of data. XC, HX, and YW takes responsibility for full-text evaluation and guidance, final approval of the version to be submitted. All the authors read and approved the final manuscript.

## Conflict of Interest

The authors declare that the research was conducted in the absence of any commercial or financial relationships that could be construed as a potential conflict of interest.

## Footnotes

1 https://www.ncbi.nlm.nih.gov/geo/query/acc.cgi?acc=GSE146763

2 http://eggnogdb.embl.de/

3 https://string-db.org/

4 https://www.ncbi.nlm.nih.gov/geo/query/acc.cgi?acc=GSE77798

5 https://www.r-project.org/

## Supplementary Material

Supplementary Table 1 The clinical features of three patients with hypoplastic left heart syndrome.

## Abbreviations

HLH: hypoplastic left heart
scRNA-seq: single-cell RNA sequencing
iPSCs: induced pluripotent stem cells
HLH-NHF: HLH without heart failure
HLH-HF: HLH with heart failure
DEproteins: differentially expressed proteins
LV: left ventricle
RV: right ventricle
GEO: Gene Expression Omnibus
t-SNE: t-Distributed Stochastic Neighbor Embedding
FC: fold change
GO: Gene Ontology
KEGG: Kyoto Encyclopedia of Genes and Genomes
DIA: data-independent acquisition
FDR: false discovery rate
PPI: protein–protein interaction
MA/AA: mitral and aortic atresia
MS/AS: mitral and aortic stenosis
PH: pulmonary hypertension
LAD: left atrium diameter
LVDD: left ventricular end-diastolic diameter
LVDS: left ventricular end-systolic diameter
RVD: right ventricle diameter
AOD: aortic diameter
2-DE: two-dimensional echocardiogram
ECG: electrocardiogram
EACTS: The European Association for Cardio-Thoracic Surgery
VSD: ventricular septal defects
ASD: atrial septal defects
DORV: double outlet right ventricle
ACCF: American Heart Association Foundation
AHA: American Heart Association.

## References

[B1] Alphonso N. Angelini A. Barron D. J. Bellsham-Revell H. Blom N. A. Brown K. (2020). Guidelines for the management of neonates and infants with hypoplastic left heart syndrome: The European Association for Cardio-Thoracic Surgery (EACTS) and the Association for European Paediatric and Congenital Cardiology (AEPC) Hypoplastic Left Heart Syndrome Guidelines Task Force. *Eur. J. Cardiothorac. Surg.* 58 416–499. 10.1093/ejcts/ezaa188 32856064

[B2] Andreasová T. Vránová J. A.-O. Vondráková D. Sedláčková L. Zákostelská Z. J. Neužil P. (2020). Role of biomarkers of cardiac remodeling, myofibrosis, and inflammation in assessment of disease severity in euvolemic patients with chronic stable heart failure. *J. Int. Med. Res.* 48:300060520947869. 10.1177/0300060520947869 32815444 PMC7444138

[B3] Astor B. C. Shafi T. Fau-Hoogeveen R. C. Hoogeveen R. C. Fau-Matsushita K. (2012). Novel markers of kidney function as predictors of ESRD, cardiovascular disease, and mortality in the general population. *Am. J. Kidney Dis.* 59 653–662. 10.1053/j.ajkd.2011.11.042 22305758 PMC3880682

[B4] Barrett T. Wilhite Se Fau-Ledoux P. Ledoux P. Fau-Evangelista C. Evangelista C. (2013). NCBI GEO: archive for functional genomics data sets–update. *Nucleic Acids Res.* 41 D991–D995. 10.1093/nar/gks1193 23193258 PMC3531084

[B5] Barron D. J. Kilby M. D. Davies B. Wright J. G. Jones T. J. Brawn W. J. (2009). Hypoplastic left heart syndrome. *Lancet* 374 551–564. 10.1016/s0140-6736(09)60563-8 19683641

[B6] Bharati S. Fau-Lev M. Lev M. (1984). The surgical anatomy of hypoplasia of aortic tract complex. *J. Thorac. Cardiovasc. Surg.* 88 97–101. 6234437

[B7] Bittle G. J. Morales D. Deatrick K. B. Parchment N. Saha P. Mishra R. (2018). Stem Cell Therapy for Hypoplastic Left Heart Syndrome: Mechanism, Clinical Application, and Future Directions. *Circ. Res.* 123 288–300. 10.1161/circresaha.117.311206 29976693 PMC6397624

[B8] Carrillo S. A. Texter K. M. Phelps C. Tan Y. McConnell P. I. Galantowicz M. (2020). Tricuspid Valve and Right Ventricular Function Throughout the Hybrid Palliation Strategy for Hypoplastic Left Heart Syndrome and Variants. *World J. Pediatr. Congenit. Heart Surg.* 12:2150135120947692. 10.1177/2150135120947692 32783502

[B9] Chaudhry F. Isherwood J. Bawa T. Patel D. Gurdziel K. Lanfear D. E. (2019). Single-Cell RNA Sequencing of the Cardiovascular System: New Looks for Old Diseases. *Front. Cardiovasc. Med.* 6:173. 10.3389/fcvm.2019.00173 31921894 PMC6914766

[B10] Crucean A. Alqahtani A. Barron D. J. Brawn W. J. Richardson R. V. O’Sullivan J. (2017). Re-evaluation of hypoplastic left heart syndrome from a developmental and morphological perspective. *Orphanet. J. Rare Dis.* 12:138. 10.1186/s13023-017-0683-4 28793912 PMC5551014

[B11] Demichev V. Messner C. B. Vernardis S. I. Lilley K. S. Ralser M. (2020). DIA-NN: neural networks and interference correction enable deep proteome coverage in high throughput. *Nat. Methods* 17 41–44. 10.1038/s41592-019-0638-x 31768060 PMC6949130

[B12] DennisG.Jr.ShermanB. T.Fau-HosackD. A.Hosack Da Fau - YangJ.Yang J Fau - GaoW. (2003). DAVID: Database for Annotation, Visualization, and Integrated Discovery. *Genome Biol.* 4:3.12734009

[B13] Egbe A. Uppu S. Lee S. Stroustrup A. Ho D. Srivastava S. (2015). Temporal variation of birth prevalence of congenital heart disease in the United States. *Congenit. Heart Dis.* 10 43–50. 10.1111/chd.12176 24612877

[B14] Elliott D. A. Kirk E. P. Yeoh T. Chandar S. McKenzie F. Taylor P. (2003). Cardiac homeobox gene NKX2-5 mutations and congenital heart disease: associations with atrial septal defect and hypoplastic left heart syndrome. *J. Am. Coll. Cardiol.* 41 2072–2076. 10.1016/s0735-1097(03)00420-0 12798584

[B15] Everitt M. D. Boyle G. J. Schechtman K. B. Zheng J. Bullock E. A. Kaza A. K. (2012). Early survival after heart transplant in young infants is lowest after failed single-ventricle palliation: a multi-institutional study. *J. Heart Lung. Transpl.* 31 509–516. 10.1016/j.healun.2011.12.013 22325692

[B16] Fixler D. E. Nembhard W. N. Salemi J. L. Ethen M. K. Canfield M. A. (2010). Mortality in first 5 years in infants with functional single ventricle born in Texas, 1996 to 2003. *Circulation* 121 644–650. 10.1161/circulationaha.109.881904 20100974

[B17] George J. Patal S. Fau-Wexler D. Wexler D. Fau-Roth A. (2005). Circulating matrix metalloproteinase-2 but not matrix metalloproteinase-3, matrix metalloproteinase-9, or tissue inhibitor of metalloproteinase-1 predicts outcome in patients with congestive heart failure. *Am. Heart J.* 150 484–487. 10.1016/j.ahj.2004.11.016 16169329

[B18] Gillet L. C. Navarro P. Tate S. Röst H. Selevsek N. Reiter L. (2012). Targeted data extraction of the MS/MS spectra generated by data-independent acquisition: a new concept for consistent and accurate proteome analysis. *Mol. Cell Proteom.* 11:016717. 10.1074/mcp.O111.016717 22261725 PMC3433915

[B19] Glidewell S. C. Miyamoto S. D. Grossfeld P. D. Clouthier D. E. Coldren C. D. Stearman R. S. (2015). Transcriptional Impact of Rare and Private Copy Number Variants in Hypoplastic Left Heart Syndrome. *Clin. Transl. Sci.* 8 682–689. 10.1111/cts.12340 26534787 PMC4703543

[B20] Gordon B. M. Rodriguez S. Lee M. Chang R. K. (2008). Decreasing number of deaths of infants with hypoplastic left heart syndrome. *J. Pediatr.* 153 354–358. 10.1016/j.jpeds.2008.03.009 18534240

[B21] Grund A. Szaroszyk M. Korf-Klingebiel M. Malek Mohammadi M. Trogisch F. A. Schrameck U. (2019). TIP30 counteracts cardiac hypertrophy and failure by inhibiting translational elongation. *EMBO Mol. Med.* 11:e10018. 10.15252/emmm.201810018 31468715 PMC6783653

[B22] Hafemeister C. Satija R. A.-O. (2019). Normalization and variance stabilization of single-cell RNA-seq data using regularized negative binomial regression. *Genome Biol.* 20:296. 10.1186/s13059-019-1874-1 31870423 PMC6927181

[B23] Hinton R. B. Martin L. J. Rame-Gowda S. Tabangin M. E. Cripe L. H. Benson D. W. (2009). Hypoplastic left heart syndrome links to chromosomes 10q and 6q and is genetically related to bicuspid aortic valve. *J. Am. Coll. Cardiol.* 53 1065–1071. 10.1016/j.jacc.2008.12.023 19298921 PMC2703749

[B24] Hoffman J. I. Kaplan S. (2006). The incidence of congenital heart disease. *Cardiol. Young* 16 339–368. 10.1017/S1047951106000291 16839428

[B25] Hrstka S. C. Li X. Nelson T. J. (2017). NOTCH1-Dependent Nitric Oxide Signaling Deficiency in Hypoplastic Left Heart Syndrome Revealed Through Patient-Specific Phenotypes Detected in Bioengineered Cardiogenesis. *Stem Cells* 35 1106–1119. 10.1002/stem.2582 28142228

[B26] Jiang Y. Habibollah S. Tilgner K. Collin J. Barta T. Al-Aama J. Y. (2014). An induced pluripotent stem cell model of hypoplastic left heart syndrome (HLH) reveals multiple expression and functional differences in HLH-derived cardiac myocytes. *Stem Cells Transl. Med.* 3 416–423. 10.5966/sctm.2013-0105 24591732 PMC3973710

[B27] Kido T. Hoashi T. Kitano M. Shimada M. Kurosaki K. Ishibashi-Ueda H. (2018). Impact of Hybrid Stage 1 Palliation for Hypoplastic Left Heart Syndrome: Histopathological Findings. *Pediatr. Cardiol.* 39 1001–1008. 10.1007/s00246-018-1851-6 29523921

[B28] Kim M. S. Fleres B. Lovett J. Anfinson M. Samudrala S. S. K. Kelly L. J. (2020). Contractility of Induced Pluripotent Stem Cell-Cardiomyocytes With an MYH6 Head Domain Variant Associated With Hypoplastic Left Heart Syndrome. *Front. Cell. Dev. Biol.* 8:440. 10.3389/fcell.2020.00440 32656206 PMC7324479

[B29] Liu X. Yagi H. A.-O. Saeed S. Bais A. S. Gabriel G. C. Chen Z. (2017). The complex genetics of hypoplastic left heart syndrome. *Nat. Genet.* 49 1152–1159. 10.1038/ng.3870 28530678 PMC5737968

[B30] Martin L. J. Ramachandran V. Cripe L. H. Hinton R. B. Andelfinger G. Tabangin M. (2007). Evidence in favor of linkage to human chromosomal regions 18q, 5q and 13q for bicuspid aortic valve and associated cardiovascular malformations. *Hum. Genet.* 121 275–284. 10.1007/s00439-006-0316-9 17203300

[B31] McBride K. L. Zender G. A. Fitzgerald-Butt S. M. Koehler D. Menesses-Diaz A. Fernbach S. (2009). Linkage analysis of left ventricular outflow tract malformations (aortic valve stenosis, coarctation of the aorta, and hypoplastic left heart syndrome). *Eur. J. Hum. Genet.* 17 811–819. 10.1038/ejhg.2008.255 19142209 PMC2916734

[B32] Miao Y. Tian L. Martin M. Paige S. L. Galdos F. X. Li J. (2020). Intrinsic Endocardial Defects Contribute to Hypoplastic Left Heart Syndrome. *Cell. Stem. Cell.* 2020:15. 10.1016/j.stem.2020.07.015 PMC7541479 32810435

[B33] Motiwala S. R. (2019). From Proteomics to Therapeutics: Sex Differences in Cardiovascular Disease Risk Do Matter. *J. Am. Coll. Cardiol.* 74 1554–1556. 10.1016/j.jacc.2019.08.011 31537264

[B34] Nakano S. J. Siomos A. K. Garcia A. M. Nguyen H. SooHoo M. Galambos C. (2017). Fibrosis-Related Gene Expression in Single Ventricle Heart Disease. *J. Pediatr.* 191 82–90.e. 10.1016/j.jpeds.2017.08.055 29050751 PMC5705574

[B35] Paige S. L. Galdos F. X. Lee S. Chin E. T. Ranjbarvaziri S. Feyen D. A. M. (2020). Patient-Specific Induced Pluripotent Stem Cells Implicate Intrinsic Impaired Contractility in Hypoplastic Left Heart Syndrome. *Circulation* 142 1605–1608. 10.1161/CIRCULATIONAHA.119.045317 33074758 PMC7583658

[B36] Radosinska J. Barancik M. Vrbjar N. (2017). Heart failure and role of circulating MMP-2 and MMP-9. *Panminerva. Med.* 59 241–253. 10.23736/S0031-0808.17.03321-3 28399617

[B37] Ramirez T. A. Saux C. Fau-Joy A. Joy A. (2012). Chronic and intermittent hypoxia differentially regulate left ventricular inflammatory and extracellular matrix responses. *Hypertens Res.* 35 811–818. 10.1038/hr.2012.32 22495609 PMC3419973

[B38] Reller M. D. Strickland M. J. Riehle-Colarusso T. Mahle W. T. Correa A. (2008). Prevalence of congenital heart defects in metropolitan Atlanta, 1998-2005. *J. Pediatr.* 153 807–813. 10.1016/j.jpeds.2008.05.059 18657826 PMC2613036

[B39] Remmell-Dow D. R. Bharati S. Fau-Davis J. T. Davis J. T. Fau-Lev M. Lev M. (1995). Hypoplasia of the eustachian valve and abnormal orientation of the limbus of the foramen ovale in hypoplastic left heart syndrome. *Am. Heart J.* 130 148–152. 10.1016/0002-8703(95)90250-3 7611106

[B40] Richer J. Hill H. L. Wang Y. Yang M. L. Hunker K. L. Lane J. (2020). Novel Recurrent COL5A1 Genetic Variant Is Associated With a Dysplasia-Associated Arterial Disease Exhibiting Dissections and Fibromuscular Dysplasia. *Arteriosc. Thromb. Vasc. Biol.* 40:Atvbaha119313885. 10.1161/atvbaha.119.313885 32938213 PMC7953329

[B41] Roulet M. Ruggiero F. Fau-Karsenty G. Karsenty G. Fau-LeGuellec D. LeGuellec D. (2007). A comprehensive study of the spatial and temporal expression of the col5a1 gene in mouse embryos: a clue for understanding collagen V function in developing connective tissues. *Cell. Tissue Res.* 327 323–332. 10.1007/s00441-006-0294-1 17024418

[B42] Saraf A. Book W. M. Nelson T. J. Xu C. (2019). Hypoplastic left heart syndrome: From bedside to bench and back. *J. Mol. Cell. Cardiol.* 135 109–118. 10.1016/j.yjmcc.2019.08.005 31419439 PMC10831616

[B43] Skelly D. A. Squiers G. T. McLellan M. A. Bolisetty M. T. Robson P. Rosenthal N. A. (2018). Single-Cell Transcriptional Profiling Reveals Cellular Diversity and Intercommunication in the Mouse Heart. *Cell Rep.* 22 600–610. 10.1016/j.celrep.2017.12.072 29346760

[B44] Stephens E. H. Gupta D. Bleiweis M. Backer C. L. Anderson R. H. Spicer D. E. (2020a). Coronary Arterial Abnormalities in Hypoplastic Left Heart Syndrome: Pathologic Characteristics of Archived Specimens. *Semin. Thorac. Cardiovasc. Surg.* 32 531–538. 10.1053/j.semtcvs.2020.02.007 32060012

[B45] Stephens E. H. Gupta D. Bleiweis M. Backer C. L. Anderson R. H. Spicer D. E. (2020b). Pathologic Characteristics of 119 Archived Specimens Showing the Phenotypic Features of Hypoplastic Left Heart Syndrome. *Semin. Thorac. Cardiovasc. Surg.* 32 895–903. 10.1053/j.semtcvs.2020.02.019 32092382

[B46] Sucharov C. C. Sucharov J. Karimpour-Fard A. Nunley K. Stauffer B. L. Miyamoto S. D. (2015). Micro-RNA expression in hypoplastic left heart syndrome. *J. Card. Fail* 21 83–88. 10.1016/j.cardfail.2014.09.013 25291457 PMC4276459

[B47] Szklarczyk D. Morris J. H. Cook H. Kuhn M. Wyder S. Simonovic M. (2017). The STRING database in 2017: quality-controlled protein-protein association networks, made broadly accessible. *Nucleic Acids Res.* 45 D362–D368. 10.1093/nar/gkw937 27924014 PMC5210637

[B48] Tchervenkov C. I. Jacobs J. P. Weinberg P. M. Aiello V. D. Béland M. J. Colan S. D. (2006). The nomenclature, definition and classification of hypoplastic left heart syndrome. *J. Am. Coll. Cardiol.* 16 339–368. 10.1017/S1047951106000291 16839428

[B49] Vianello A. Caponi L. Galetta F. Franzoni F. Taddei M. Rossi M. (2015). β2-Microglobulin and TIMP1 Are Linked Together in Cardiorenal Remodeling and Failure. *Cardiorenal. Med.* 5 1–11. 10.1159/000369260 25759695 PMC4327333

[B50] Wang H. J. Si Q. J. Shi Y. Guo Y. Li Y. Wang Y. T. (2018). The prognostic values of beta-2 microglobulin for risks of cardiovascular events and mortality in the elderly patients with isolated systolic hypertension. *J. Res. Med. Sci.* 23:82. 10.4103/jrms.JRMS_135_17 PMC6161488 30294350

[B51] Weerakkody R. A. Vandrovcova J. Kanonidou C. Mueller M. Gampawar P. Ibrahim Y. (2016). Targeted next-generation sequencing makes new molecular diagnoses and expands genotype-phenotype relationship in Ehlers-Danlos syndrome. *Genet. Med.* 18 1119–1127. 10.1038/gim.2016.14 27011056

[B52] Yagi H. Liu X. Gabriel G. C. Wu Y. Peterson K. Murray S. A. (2018). The Genetic Landscape of Hypoplastic Left Heart Syndrome. *Pediatr. Cardiol.* 39 1069–1081. 10.1007/s00246-018-1861-4 29569026 PMC8565805

[B53] Yokota T. McCourt J. Ma F. Ren S. Li S. Kim T. H. (2020). Type V Collagen in Scar Tissue Regulates the Size of Scar after Heart Injury. *Cell* 182 545–562.e. 10.1016/j.cell.2020.06.030 32621799 PMC7415659

[B54] Yu G. Wang Lg Fau-Han Y. Han Y. Fau-He Q.-Y. He Q. Y. (2012). clusterProfiler: an R package for comparing biological themes among gene clusters. *OMICS* 16 284–287. 10.1089/omi.2011.0118 22455463 PMC3339379

