# Supplementary material for: Single-Cell RNA Sequencing and Quantitative Proteomics Analysis Elucidate Marker Genes and Molecular Mechanisms in Hypoplastic Left Heart Patients With Heart Failure
Source: Front Cell Dev Biol. 2021 Feb 25;9:617853. doi: 10.3389/fcell.2021.617853 (PMC7946977; doi:10.3389/fcell.2021.617853)
Supplement: Supplementary Table 1 — The clinical features of three patients with hypoplastic left heart syndrome. [file Data_Sheet_1.PDF]

**Table S1. The clinical features of three patients with hypoplastic left heart syndrome.**

**Patient 1:** Control: There was no congenital heart malformation and heart failure.

A 2-months-old baby boy was born at Guangzhou Women and Children's Medical Center. At birth, the Apgar scores were 10 ,10 and 10 at 1, 5 and 10 min, respectively. Birth weight was 3.1 kg. He had been hospitalization for one week for primary viral pneumonia. Results of N-terminal pro-brain natriuretic peptide (NT-proBNP), Brain natriuretic peptide (BNP), physical examination and echocardiography were normal.

**(Without the 3D reconstruction of cardiac CT)**

**Patient 2:** HLHS-HF: A 1 Months-old baby boy was referred to Guangzhou Women and Children's Medical Center on December 12, 2019 from Hunan Children Hospital with a diagnosis of ventricular septal defect. At birth, the Apgar scores were 9 ,8 and 8 at 1, 5 and 10 min, respectively. Birth weight was 3.4 kg. He was treated for recurrent pneumonia with congestive cardiac failure on three occasions with cefuroxime and digoxin in a community clinic center with some relief of symptoms before being referred due to worsening of symptoms. Physical examination revealed the central and peripheral cyanosis, no peripheral edema. oxygen saturations of 85% and a heart rate of 170 beats/min. Blood pressure in the right arm and in the lower limb were 62/35 and 62/38 mmHg, respectively. The first and second heart sounds were heard, they were of normal intensity but with a pansystolic murmur grade 3~4/6 maximal at the left lower sternal edge. Chest X-ray revealed gross cardiomegaly with plethora of lung fields. The echocardiography and cardiac CT showed that the right ventricle was enlarged, the wall of right ventricle was thickened, and the left ventricle was small. BNP was 768 pg/ml, NT pro-BNP was 1025 pg/ml.

**3D reconstruction of cardiac CT:**

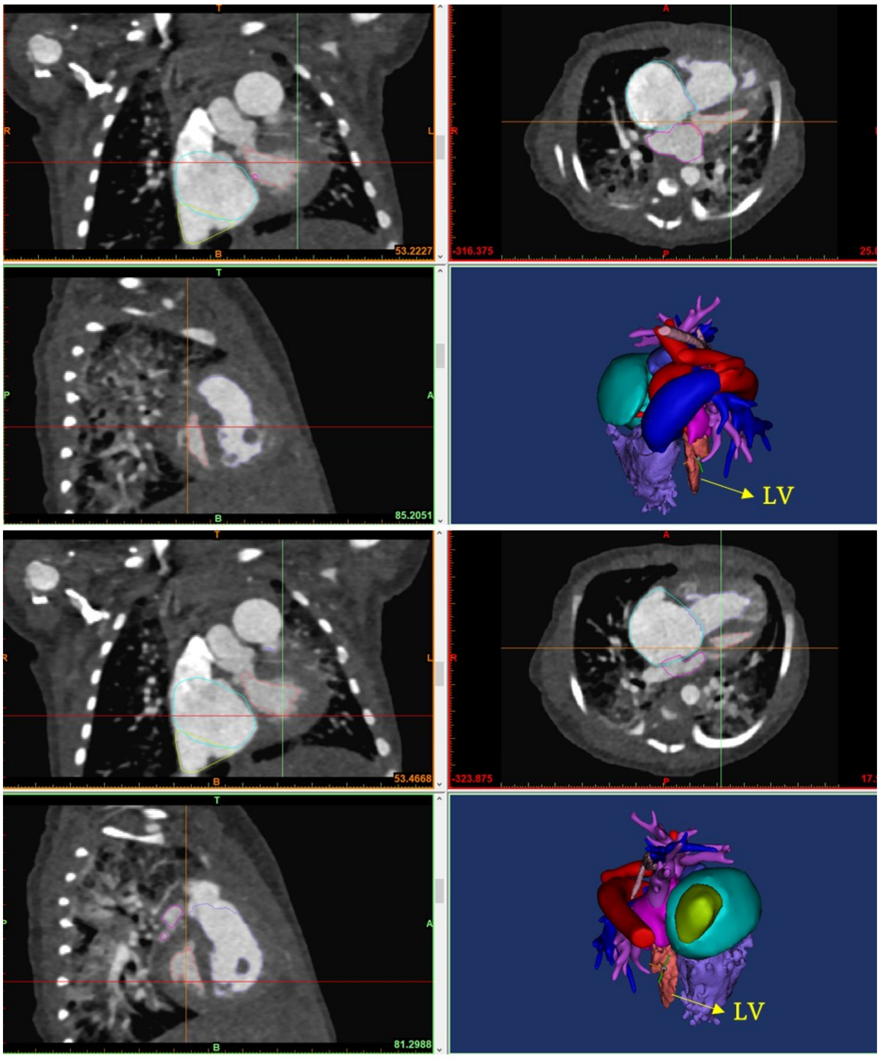


**Patient 3:** HLHS-NHF: A 6 Weeks-old baby boy was born at Guangzhou Women and Children's Medical Center. At birth, the Apgar scores were 8 ,8 and 8 at 1, 5 and 10 min, respectively. Birth weight was 2.9 kg. Here, a loud murmur was heard on examination after birth, for which an echocardiogram was performed. Physical examination revealed oxygen saturations of 86% and a heart rate of 180 beats/min. Blood pressure in the right arm and in the lower limb were 62/36 and 66/38 mmHg, respectively. The normal first heart sound, single second heart sound, systolic ejection click, systolic ejection murmur grade 3~4/6 at the upper left sternal border, and absence of a diastolic murmur. Chest X-ray revealed gross cardiomegaly. The echocardiography and cardiac CT showed that the right ventricle was enlarged, the wall of right ventricle was thickened, and the left ventricle was small. BNP was 64 pg/ml, NT pro-BNP was 105 pg/ml.

**3D reconstruction of cardiac CT:**

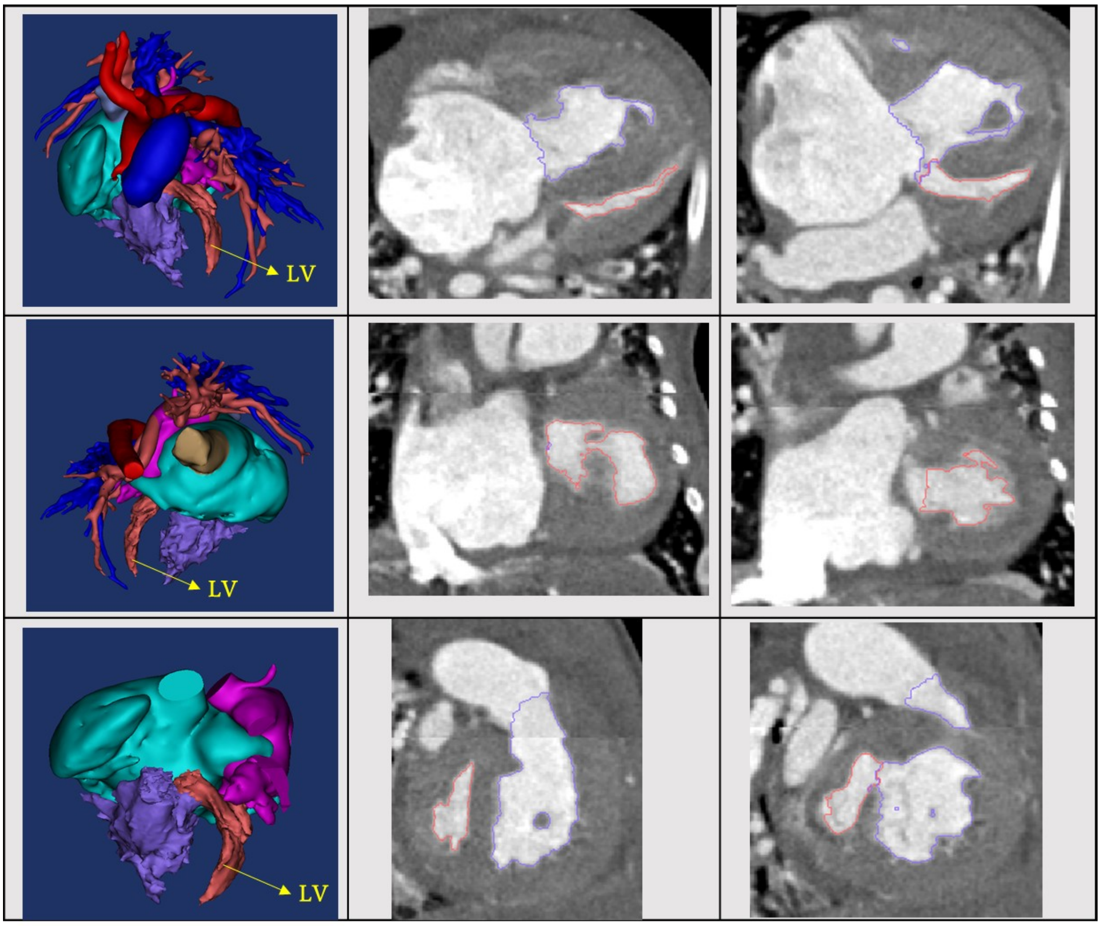
LV
LV
LV
